# Electrochemical Impedance Spectroscopy-Based Sensing of Biofilms: A Comprehensive Review

**DOI:** 10.3390/bios13080777

**Published:** 2023-07-31

**Authors:** Sikander Ameer, Hussam Ibrahim, Muhammad Usama Yaseen, Fnu Kulsoom, Stefano Cinti, Mazhar Sher

**Affiliations:** 1 Department of Agricultural and Biosystems Engineering, South Dakota State University, Brookings, SD 57007, USA; 2 Department of Electrical & Computer Engineering, Iowa State University, Ames, IA 50011, USA; 3 Department of Biosystems and Agricultural Engineering, Oklahoma State University, Stillwater, OK 74078, USA; 4 Department of Zoology, Abbottabad University of Science and Technology, Havelian 22500, Pakistan; 5 Department of Pharmacy, University of Naples “Federico II”, Via D. Montesano 49, 80131 Naples, Italy; 6 BAT Center-Interuniversity Center for Studies on Bioinspired Agro-Environmental Technology, University of Napoli “Federico II”, 80055 Naples, Italy

**Keywords:** biofilms, electrochemical impedance spectroscopy, biosensors

## Abstract

Biofilms are complex communities of microorganisms that can form on various surfaces, including medical devices, industrial equipment, and natural environments. The presence of biofilms can lead to a range of problems, including infections, reduced efficiency and failure of equipment, biofouling or spoilage, and environmental damage. As a result, there is a growing need for tools to measure and monitor levels of biofilms in various biomedical, pharmaceutical, and food processing settings. In recent years, electrochemical impedance sensing has emerged as a promising approach for real-time, non-destructive, and rapid monitoring of biofilms. This article sheds light on electrochemical sensing for measuring biofilms, including its high sensitivity, non-destructive nature, versatility, low cost, and real-time monitoring capabilities. We also discussed some electrochemical sensing applications for studying biofilms in medical, environmental, and industrial settings. This article also presents future perspectives for research that would lead to the creation of reliable, quick, easy-to-use biosensors mounted on unmanned aerial vehicles (UAVs), and unmanned ground vehicles (UGVs), utilizing artificial intelligence-based terminologies to detect biofilms.

## 1. Introduction

Despite more than 40 years of research on microbial biofilms, it is still challenging to treat biofilm infections. Environmental biofilms create substantial annual economic losses that are estimated to be around $5 trillion worldwide and are a potential source of pathogens and genes for antibiotic resistance [1,2]. Biofilm causes different types of human diseases e.g., cystic fibrosis, tuberculosis, orthodontal disease, sinusitis, and some forms of heart disease [3]. It can also be a reason for food contamination during processing and packaging. In nature, the production of biofilms is an essential aspect of the bacterial life cycle. Where the food is processed, cross-contamination during unhygienic food preparation methods and raw or undercooked food consumption are the main sources of bacterial transmission. As a means of surviving in a variety of unfavorable conditions, foodborne bacteria create biofilms, which are frequently a cause of repeated infection and outbreaks of foodborne diseases [4]. Biofilm on food contact surface in restaurants and home kitchens provide foodborne pathogens such as salmonella and *E. coli* the opportunity to survive [5]. The water supply pipes could also be affected by biofilms. Dental plaque is a common form of biofilm, a typical biofilm made up of a complicated microbial community [6]. There are 12.5 billion tons (US) of bacteria in the 50 million km^2^ of managed soils around the world, which is equivalent to 2.6 × 10^29^ bacterial cells [7]. The generation of food from both plants and animals is impacted by a variety of crucial processes in which these bacteria are involved. The management and control of biofilms is essential for guaranteeing effective food production and food safety throughout the cycle of food creation, handling, and delivery, both in agriculture and in the supply chain. Biofilms are also an essential part of $3700 bn global agricultural activity [2,8] and it is predicted that these biofilms will have a $324 bn annual global impact. To support agricultural productivity, there is a rising importance in managing biofilms and microbiomes [2].

Biofilms and microbiomes play critical roles in the agriculture industry [9,10]. Biofilms are communities of microorganisms that attach to surfaces, including those found in soil and on plant roots. These biofilms can either positively or negatively impact agricultural productivity [11,12,13].

In some cases, biofilms can benefit plants by promoting nutrient uptake and protecting against pathogens. For example, some biofilm bacteria can form symbiotic relationships with plants, providing them with essential nutrients such as nitrogen. In other cases, biofilms can cause harm by promoting the growth of pathogenic microorganisms that can damage crops [11,12,13,14].

Microbiomes, communities of microorganisms found in soil, water, and plants, are also essential for agricultural productivity. Microbiomes can help to promote plant growth by providing nutrients, protecting against pathogens, and promoting soil health.

Given the importance of biofilms and microbiomes in agriculture, there is a growing interest in managing and quantifying them to support agricultural productivity. This can involve using beneficial microorganisms to promote plant growth and antimicrobial agents to control harmful biofilms [15]. By better understanding and managing these microbial communities, it may be possible to increase agricultural productivity and sustainability while also reducing the use of harmful chemicals [16,17].

A biofilm refers to a collection of microorganisms colonized in a single matrix. Extracellular polymeric substance (EPS) matrix of surface-associated microbial cells known as biofilms. Van Leeuwenhoek is known for discovering microbial biofilms after using a simple microscope to detect bacteria on tooth surfaces. In 1940, Heukelekian and Heller focused on the ‘bottle effect’ of marine microorganisms, where they studied how the presence of surfaces on which these organisms stick can greatly increase bacterial activity and growth [18]. The material named extracellular polymer substance (EPS) is used for making the matrix. A biofilm weighing 1 g contains 10^8^ to 10^11^ cells. The same bacteria can behave differently in biofilms than in a free-living state (plankton) [19]. A group of microbial cells forms a biofilm surrounded with a polysaccharide-based matrix that is permanently attached to a surface (cannot be removed by rinsing). The entire biofilm species is covered by the cell’s outer layer and thick extracellular matrix, which also increases the biofilm’s resilience to detergents and antibiotics. It disrupts the use of antibiotics. The bacteria in biofilm are determined to be a thousand times more resistant to antibiotics as compared to the individual bacteria. The only option for treatment is to remove the contaminated implant if the antibiotic therapy is insufficient.

Non-cellular substances, for example, crystallized minerals, blood, silt, or clay elements may be included in the biofilm, based upon the environment where the biofilm is developed. Genes that are transcribed differently in biofilm-associated organisms than in planktonic (freely suspended) organisms. Biofilms can occur on different surfaces, including living tissue, pipelines in industrial or drinking water systems, and aquatic ecosystems. In the industry, biofilms have also led to several issues. It can pollute commodities, contaminate equipment, and harm the distribution system for water. Due to the biofilm bacteria’s emission of H_2_S, it can result in fuel contamination and chemical souring.

**Figure 1:**
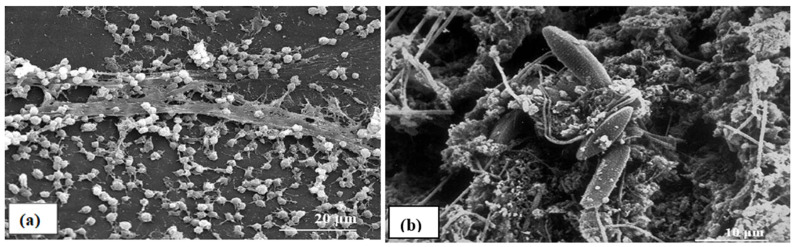
(Reprinted from [20] under the Creative Commons Attribution 4.0 International Public License https://creativecommons.org/licenses/by/4.0/legalcode (accessed on 20 June 2023)) (**a**) Biofilm on an indwelling medical device. (**b**) Biofilm on mild steel surface developed after 8 weeks.

Scanning electron micrographs of biofilms from indwelling medical devices and industrial water systems respectively shown in Figure 1. Biofilms are found everywhere in the environment. It can be found in noses, shower curtains, stones in rivers, streams, and surgical equipment. According to the CDC, biofilms are to blame for 65 percent of nosocomial infections. The body’s implanted medical devices may be impacted by the development of biofilm. Catheters, artificial joints, and mechanical heart valves are some examples of these implanted devices. Microorganism colonization can lead to a gradual and permanent infection. Highly complex aqueous biofilms formed from filamentous bacteria, clay particles, freshwater diatoms, and from corrosion results. In contrast, medical device biofilms seem to be made up of a single bacterium and connected with an extracellular polymeric substance (EPS) matrix [5].

One example of a tool used to measure levels of biofilms is the crystal violet assay, a simple and widely used method for quantifying biofilm formation. In this assay, bacteria are grown on a surface in a nutrient-rich medium, and the resulting biofilm is stained with crystal violet. The amount of crystal violet that adheres to the biofilm is then measured, which provides a quantitative measure of biofilm formation [21].

Another example of a tool used to measure biofilms is confocal laser scanning microscopy (CLSM), which allows for visualization of the three-dimensional structure of the biofilm. In CLSM, a laser excites fluorescent dyes or proteins within the biofilm, generating a three-dimensional image of the biofilm structure. This technique can provide information on the biofilm’s thickness, porosity, and spatial distribution, which can help researchers better understand the factors that contribute to biofilm formation and growth [22].

Electrochemical sensing is another approach used to measure biofilms. This technique involves using electrodes to detect the electrochemical signals produced by the biofilm. The electrochemical signals can provide information about the metabolic activity and composition of the biofilm. Electrochemical sensing can be used to monitor biofilms in real-time and has the potential to provide a rapid and sensitive method for detecting and monitoring biofilms in a variety of settings, including in medical and industrial applications.

Electrochemical sensing offers several advantages for monitoring biofilms. First, electrochemical sensors can provide real-time monitoring of biofilm growth and activity, which allows for rapid detection of changes in biofilm levels or characteristics. This is particularly useful in settings where rapid detection and response are critical, such as in medical or industrial applications. Secondly, electrochemical sensing offers a great solution to detect low-concentration analytes due to their operation principle; it involves electron transfer processes, allowing for the directly proportional relationship between analyte concentration and the resulting electrical signal, enabling precise quantification of low analyte concentrations. This is important for detecting biofilms in the early stages of growth before they become established and more difficult to remove. Third, electrochemical sensing is a non-destructive method for monitoring biofilms; the biofilm remains intact and can continue to grow and develop. This is important for monitoring biofilms over time, as it allows for repeated measurements without disrupting the biofilm structure. Fourth, electrochemical sensing can be used to monitor a wide range of parameters related to biofilm growth and activity, including metabolic activity, cell density, and changes in pH or other environmental factors. Such advantage makes electrochemical sensing a versatile tool for studying biofilms in different settings and under different conditions. Finally, electrochemical sensors can be relatively inexpensive to produce and use, especially compared to previous methods discussed for monitoring biofilms.

**Figure 2:**
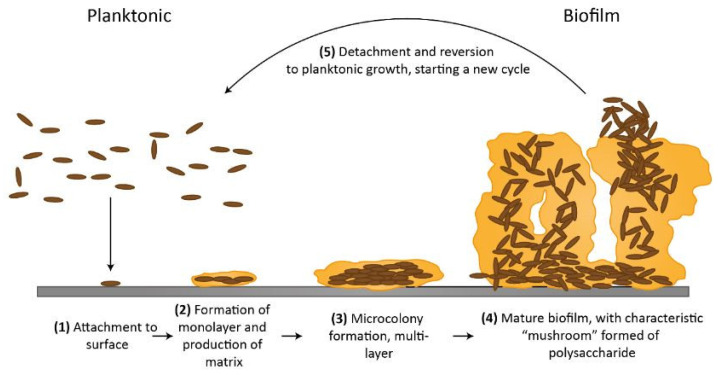
(Reprinted from [25]. under the Creative Commons Attribution 4.0 International Public License https://creativecommons.org/licenses/by/4.0/legalcode (accessed on 20 June 2023)) Representation of flow diagram of biofilm formation.

The production of biofilms is a dynamic phenomenon [23,24]. Planktonic bacteria can join forces with other organisms to create a complex biofilm when it is attached to a surface. Every organism has a special way of attaching to objects. Attachment by pili, flagella, proteins, and polysaccharide adhesins are a few of the methods [23]. The process of biofilm can be divided into five steps; these steps are shown in the flow chart in Figure 2. These five processes are known as (1) initial reversible attachment, (2 and 3) irreversible attachment, (4) maturation and (5) dispersion. At first, this stage is reversible and occurs when the migrating planktonic bacteria come into initial contact with the surface. 

After this, a monolayer of bacteria will start to form and create an extracellular matrix, sometimes known as “slime”. Extracellular polymeric substances (EPS), also known as extracellular polysaccharides having structural proteins, nucleic acids, and cell detritus produce the matrix. Extracellular DNA (eDNA) controls the initial phases of matrix creation, while structural proteins and polysaccharides come afterwards. These phases result in the creation of microcolonies, which show notable development and cell-cell interactions including quorum sensing. The bond of biofilm is now irrevocable as biofilm develops in three dimensions [26].

More than 90% of the dry mass of biofilms is composed of the biofilm matrix, which offers the bacteria a three-dimensional microenvironment to keep them safe [27]. This structure, which is a distinctive characteristic that distinguishes biofilms, closely regulates the functional and physical characteristics of biofilm [28]. The structure of the extracellular polymeric substances (EPS) in the matrix, such as nucleic acids, lipids, proteins, and polysaccharides, as well as the physical structure of the biofilm, differ between bacterial species [29]. 

In the last stage, a few mature biofilm cells begin to separate and scatter into the surroundings as planktonic cells to start a fresh cycle of biofilm development [30]. The medical sciences have challenging issues with biofilm development because it interacts with standard treatments and is connected to illnesses. 

## 2. Sensing of Biofilms

Different electrochemical techniques have been developed to examine biofilm sensing throughout this broad range of uses including electrochemical impedance spectroscopy (EIS), chronopotentiometry, chronoamperometry, cyclic voltammetry, and scanning electrochemical microscopy [31,32].

An electrochemical reaction at an electrode surface can be broken down mechanistically into several steps processes (charge transfer, adsorption, and mass transport processes), each happening at a different rate. Different steps might happen at various timescales and are time-dependent. To make the evaluation of electrochemical systems easier more rapid approaches such as EIS are necessary. The EIS enables the examination of the time-dependent mechanism through the electrochemical system’s response (current or potential) recorded at specific frequencies. The EIS technique is useful because it can explain the electrochemical mechanisms taking place on the surface of the electrode in only one measurement. These mechanisms include the operating of electrochemical biosensors, the operating of industrial batteries, and the corrosion of alloys and metals.

### 2.1. Electrochemical Impedance Spectroscopy for Sensing Purpose

Electrochemical biosensors are a famous type of sensor that are commercially successful to be developed [33]. According to the operational principle and mechanism of measurement, these are generally classified into two types: impedimetric (non-faradaic) transducers and faradaic (amperometric, potentiometric). The non-faradaic electrochemical impedance spectroscopy (EIS) is a non-destructive method; thus, it allows repeated measurements on the same sample. EIS also measures the impedance over a range of frequencies, and it is highly sensitive to very small changes in the biofilm. The EIS does not require direct current. It does not involve redox reactions and there is no need for a reference electrode. As a result, the EIS-based methods for biofilm detection are more amenable to miniaturization compared to faradaic methods. To analyze EIS data, some knowledge of electrochemistry and biofilm physics is required. EIS experiments are time-consuming as multiple measurements at different frequencies are required. EIS of biofilms is well-suited for applications where highly sensitive, non-destructive, and time-dependent measurements are required. EIS is an excellent choice for studying the overall dynamic behavior of biofilms. It provides an opportunity to investigate the biofilm responses to various conditions. EIS would be the preferred choice to monitor biofilm growth over the passage of time.

The faradaic methods of biofilm detection focus on specific electrochemical reactions. Thus, they provide specific information about biofilm components. As faradaic approaches do not require multiple frequency sweeps, they provide faster measurements. Faradaic methods typically involve simpler data analysis compared to the more complex impedance spectra of EIS. The Faradic techniques often perturb the system with electrochemical reactions, which can destroy the biofilm. Destructive Faradaic approaches might not be as sensitive to rapid small changes in the biofilm as EIS. Faradaic electrochemical approaches can be utilized where non-destructive measurements are not a priority and researchers want to obtain specific information about biofilm components or processes. Faradaic approaches are less time-consuming and require simple data analysis.

The ultimate choice between EIS and faradaic electrochemical methods for biofilm detection will depend on the specific application where researchers can choose one based on their specific requirements and considering sensitivity, data complexity, and non-destructive nature. 

#### 2.1.1. Impedimetric Biosensors

To detect and quantify bacteria, impedance-based methods have been employed as a transduction mechanism. Impedance microbiology (IM) has been utilized for years to identify bacteria from the samples of the environment, food sector, healthcare, etc. In this method, a pair of electrodes is immersed in the culture media. The idea is to measure the change in impedance. Capacitance or impedance and total or relative change in conductivity of the solutions are evaluated at a specific temperature, to identify bacterial development in real-time. Direct or indirect measurement techniques are used in conventional impedance microbiology to measure the impedance change of the media. The overall impedance change due to bacterial development is composed of two elements that could be monitored at various ranges of frequency: (i) change in impedance by the media and (ii) change in impedance caused by the electrolyte/electrode interaction, typically called as the double electrochemical layer (EDL). The impedimetric procedure used to measure electrochemical impedance is typically non-faradaic. The impedance of the growing medium will become more effective on frequencies over 10 kHz, whereas the EDL impedance is more prominent at lower frequencies (usually < 10 kHz). A basic circuit involving two EDL capacitors with value *C_dl_* and a resistor *R_s_* connected in series can be utilized to explain how both impedances’ frequency impacts total impedance. Equation (1) below can be used to numerically show the circuit’s impedance Z:

where

*f* = Frequency 

*R_s_* = Solution resistance 

*C_dl_* = EDL capacitance at the electrode.

(1) $$\left|z\right|=\sqrt{R_{s}^{2}+{\left(\begin{array}{c} 1\\ \bar{\pi fC_{dl}} \end{array}\right)}^{2}}$$

**Figure 3:**
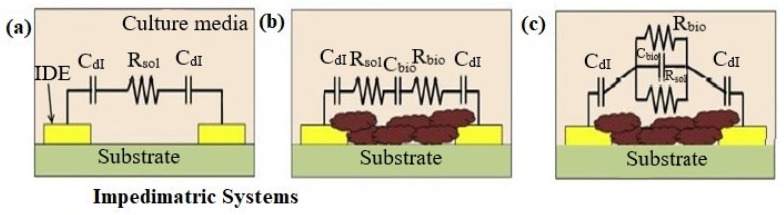
Reprinted with permission from Elsevier [35] (**a**) Model of sterile culture media before bacterial cells are inoculated. (**b**) Model of series circuits after ECM and biofilm development (**c**) models of parallel circuits after ECM and biofilm development.

**Figure 4:**
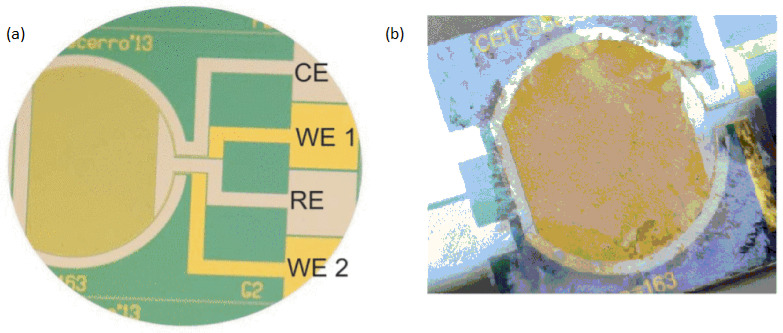
Reprinted from [35] with permission from Elsevier (**a**) An image of the thin-film electrochemical biosensor taken before biofilm growth. (**b**) The Actual image of electrochemical biosensor after the experiment of biofilm formation.

The electrochemical impedance of biofilms is produced by the extracellular matrix (ECM) and the cells inside of the biofilm act as dielectric materials, which change with metabolic state, composition, and time. It is possible to model the bacterial biofilms that have developed on the surface of microelectrodes as an electrical circuit. Figure 3a–c shows such an electronic circuit model. A sterile electrical model growth medium without bacteria is shown in Figure 3a. ECM and biofilm are produced between the two electrodes; a simple series and corresponding parallel electrical model is shown in Figure 3b,c. The following are the parameters in the circuit stand for: The EDL capacitance is denoted by the Cdl, the media resistance without bacterial cells are denoted by the *Rsol*, and the resistance and capacitance of biofilm are denoted by *Rbio* and *Cbio*, respectively. The impedimetric responses of the culture change accordingly when the first two variables Cdl and *Rsol* are affected by bacterial metabolism [34]. Equations (2)–(4) can be used to figure out the magnitude of the impedance of the three electrical circuits illustrated in Figure 4a–c.

(2) $$z_{\left(a\right)}=R_{sol}+\frac{2}{j\omega Cdl}$$

(3) $$z_{\left(b\right)}=R_{sol}+R_{bio}+\frac{2}{j\omega Cdl}+\frac{1}{j\omega Cbio}$$

(4) $$z_{\left(c\right)}=\frac{R_{sol}R_{bio}}{R_{sol}+j\omega R_{sol}R_{bio}Cdl+R_{bio}}+\frac{2}{j\omega Cdl}$$

These formulas were used to evaluate experimental data for Staphylococcus epidermis biofilms, and researchers were able to determine the numerical values of the different parameters [34]. Specific electrical properties of the system can be monitored and applied to precisely detect the initiation of biofilm development as well as the development with respect to time by applying these or other similar models to the experimental data. For instance, Liu et al. used a similar circuit model and an interdigitated microelectrodes (IDE)-based impedance sensor to monitor variations in the capacitance and resistance of Salmonella and *E. coli* biofilms with time [36]. Microelectrode arrays have also been used to measure the capacitive and resistive features of *E. coli* growth [37]. Impedimetric techniques are among the most widely used approaches for detecting and characterizing biofilms due to their benefits, which include a low resistance, good signal-to-noise ratio, decrease in sample volume, low power requirement, and quick establishment of a steady state [38].

#### 2.1.2. Potentiometric and Amperometric Biosensors

For real-time biofilm sensing, faradic electrochemical approaches including amperometric and potentiometric techniques have also been used. To measure the faradaic current produced through the oxidation and reduction of a redox species with the interaction of a solid electrode is accomplished by these measurements. It has been demonstrated that charge transmission takes place among the cells and the medium through the early phases of bacterial biofilms [39,40]. Different types of molecules produced by bacterial cells, such as pyocyanin and phenazine-1-carboxylic acid, include electrochemically active groups which can connect with the free electrons of a surface. Electrochemical methods allow for the observation of this phenomenon, their analysis, and subsequent detection of bacterial existence at the early phases of adhesive and biofilm development. Beccero et al., 2016 created a thin-film sensor [41]. It was developed by differential pulse voltammetry (DPV) and cyclic voltammetry (CV). The four-microelectrode configuration as shown in Figure 4 was used by the researchers consists of a pseudo-reference electrode made of platinum, a counter electrode made of platinum, and two gold working electrodes. In comparison to the two-electrode arrangement, this method showed high sensitivity, a faster time to steady-state current, and a smaller ohmic drop [42,43]. 

The existence of a Staphylococcus epidermidis biofilm was discovered after 2 h of the first inoculation by CV and 1 h through DPV. There was an increase in both the current signal and the redox peaks were observed proportionally to biofilm growth. 

### 2.2. Electrochemical Impedance Spectroscopy Existing Technologies

Electrochemical impedance spectroscopy is a powerful tool for the time-dependent mechanism examination of biofilms. This method provides real-time and non-destructive measurements. Hence, it is well suited to analyze biofilm dynamics at various time frames. Thus, it can be used to understand biofilm formation, growth, and response to environmental changes. Here, we present detailed information about the electrochemical impedance spectroscopy-based technologies for the detection of biofilms.

#### 2.2.1. Flexible Platform for In-Situ Impedimetric Detection

In another research, a real-time biofilm formation detection system and treatment on surfaces of 3D biomedical devices was developed [44]. They designed a flexible platform on polyimide substrates with gold-interdigitated electrodes. The characterization was carried out using a specially designed flow system, and the sensor was mounted within a urinary catheter. To monitor the formation of the biofilm a 50 mV signal amplitude impedance change was used at 100 Hz. *Escherichia coli* (*E. coli*) biofilm growth was related to a 30% reduction in impedance within 24 h. The platform allowed for biofilm removal via the bioelectric effect; a small amount of antibiotic in combination with the application of an AC voltage signal resulted in a synergistic decrease in biofilm, which caused a 12% rise in impedance. The results of the impedance detection matched with variations in the quantity of biofilm biomass on the sensor, according to biomass characterization performed with crystal violet staining. The reduction in size and allowing for on-the-go wireless implementation proved integration by using an impedance converter chip. The impedance converter decreased by 5% impedance, which simulated the potentiostat trend was connected to the development of biofilm. 

#### 2.2.2. Monitoring of Bacteria Biofilms by In-Situ Impedimetric Biosensor Chip

In another research, a chip of biosensor with interdigital microelectrodes was designed and used for observing the development of *E. coli* and Salmonella biofilms [36]. An interdigital microelectrode with a glass substrate and a PDMS layer with small cavities formed the chip of the biosensor. By applying 100 mV of alternating voltage and using a 1 Hz to 100 kHz range of frequency for 48 h, the biosensor chip monitored the EIS of *E. coli* and Salmonella biofilms. It was observed that the impedance spectroscopy of biofilms changed with culture time. Additionally, a model of an analogous circuit that considers the biofilm resistance (Rb) and capacitance (Cb) properties was used to fit the impedance spectroscopy of biofilm. The findings showed that the Cb first drops and then increases during the time of culture, but the Rb showed a vice-versa trend with respect to culture time. Additionally, it was shown that *E. coli* and Salmonella had very distinct Cb and Rb changing trends with culture time. Due to its distinct characteristics of continuity, in-situ monitoring, and non-invasion for bacteria biofilms identification and real-time, the chip of a biosensor offered a feasible platform for more research into biofilms. This biosensor chip can monitor the biofilm formation of Salmonella and *E. coli* in real-time.

#### 2.2.3. Integrated Microsystem for Real-Time Detection of Bacterial Biofilms

Subramanian et al. 2017 designed an impedance monitoring device with threshold-activated feedback for simultaneous treatment and in-situ biofilm recognition [45]. They showed how to measure the fractional relative change (FRC) in absolute impedance to properly detect the development of biofilms of *Escherichia coli* in microfluidic flow cells. Additionally, they also showed that growth measurements can be used as a threshold-activated initiation tool to start effective biofilm handling by using the bioelectric effect (BE), conducted, and using the same array of sensing electrodes. It was achieved by a customized database that (a) allowed the threshold-based activation of BE treatment and (b) provided in-situ detection of the elimination and development of biofilms inside the microfluidic channels. This developed microsystem will enable real-time detection of the onset of biofilms and their in-situ treatment on the surfaces of medical implants.

Table 1 presents the details of some other research studies for the detection of biofilms. It summarizes the important characteristics such as the limit of detection and response time of various devices used for the detection of biofilms. It includes both electrochemical and optical biosensors for the detection of biofilm. Table 2 provides information about the electrochemical devices for the detection of biofilms. 

Although EIS is a powerful tool for biofilm sensing, it does have a few limitations such as complex data analysis and electrode fouling [54].

## 3. Conclusions and Future Directions

It is a need of time to detect biofilms by using methods that are rapid, sensitive, selective, easy to use, and feasible for in-situ detection of biofilm. There are different methods to detect biofilm e.g., optical sensors, electrochemical sensors, gas sensors, E-noses, and odor-based sensing methods. In this review electrochemical impedance spectroscopy-based techniques have been discussed in detail. EIS is a powerful tool to monitor the growth of biofilms in real-time. EIS is a noninvasive, sensitive, and label-free method for the rapid monitoring of biofilms. EIS-based sensors utilize very low power and are extremely sensitive to very small changes in the environment. It is also possible to easily miniaturize the EIS-based sensors. This miniaturization can facilitate the development of microsystems for the real-time detection of biofilms in medical devices and other systems. The detection of biofilms by using electrochemical sensors would be significant in the near future by mounting these sensors on unmanned aerial vehicles (UAVs), unmanned ground vehicles (UGVs), and artificial intelligence (AI) enabled robots for hospital settings, agriculture farms, water supply lines, marine structures, and food manufacturing and processing plants. As a result, it would be possible to rapidly detect biofilms and apply quick remedial measures. Therefore, it would be a cost-effective and rapid method to minimize the losses caused by biofilms using Electrochemical Impedance Spectroscopy-based sensors.

## Figures and Tables

**Table 1:** Existing Devices for Biofilm Detection.

Sensors	Mechanism	Target	Limit of Detection (LOD)	Response Time	Reference
Electrochemical	Impedance measurements	Staphylococcus aureus	240 Bacteria cells	<10 min	[46]
Electrochemical	Impedance Measurements	Human cervical carcinoma (HeLa) cells	N/A	>20 min	[47]
Electrochemical	Impedance Measurements	*Escherichia coli*	N/A	N/A	[45]
Optical	FTIR	*E. coli* DPD2794	N/A	>40 min	[48]
Optical	MIR	Proteus mirabilis	N/A	N/A	[49]
Optical	SPR	*E. coli*	8.81 × 10^4^ cfu/mL	N/A	[50]

**Table 2:** Current Electrochemical Devices for Biofilm Detection.

Bacteria of Interest	Detection Range	Limit of Detection	Reference
*E. coli*	1.3 × 10^−18^ to 10 × 10^−12^ M	1.3 × 10^−18^ M	[51]
*E. coli*	10^−6^ to 10^−16^ M	1 × 10^−16^ M	[52]
*E. coli*	5.0 CFU mL^−1^ to 10^6^ CFU mL^−1^	3.0 CFU mL^−1^	[53]

## Data Availability

Not Applicable.
